# The KDIGO acute kidney injury guidelines for cardiac surgery patients in critical care: a validation study

**DOI:** 10.1186/s12882-018-0946-x

**Published:** 2018-06-25

**Authors:** Samuel H. Howitt, Stuart W. Grant, Camila Caiado, Eric Carlson, Dowan Kwon, Ioannis Dimarakis, Ignacio Malagon, Charles McCollum

**Affiliations:** 1 Division of Cardiovascular Sciences, University of Manchester, 2nd Floor ERC, Wythenshawe Hospital, Manchester University Hospitals Foundation Trust, M23 9LT, Manchester, UK; 2 Department of Cardiothoracic Anaesthesia and Critical Care, Wythenshawe Hospital, Manchester University Hospitals Foundation Trust, M23 9LT, Manchester, UK; 3 Department of Statistics, Durham University, Durham, DH1 3LE UK; 4 Academic Surgery Unit, ERC, Manchester University Hospitals Foundation Trust, M23 9LT, Manchester, UK; 5 Department of Cardiothoracic Surgery, Wythenshawe Hospital, Manchester University Hospitals Foundation Trust, M23 9LT, Manchester, UK

**Keywords:** Acute kidney injury, Cardiac surgery, Critical care

## Abstract

**Background:**

The Kidney Disease: Improving Global Outcomes (KDIGO) Acute Kidney Injury (AKI) guidelines assign the same stage of AKI to patients whether they fulfil urine output criteria, serum creatinine criteria or both criteria for that stage. This study explores the validity of the KDIGO guidelines as a tool to stratify the risk of adverse outcomes in cardiac surgery patients.

**Methods:**

Prospective data from consecutive adult patients admitted to the cardiac intensive care unit (CICU) following cardiac surgery between January 2013 and May 2015 were analysed. Patients were assigned to groups based on the criteria they met for each stage of AKI according to the KDIGO guidelines. Short and mid-term outcomes were compared between these groups.

**Results:**

A total of 2267 patients were included with 772 meeting criteria for AKI-1 and 222 meeting criteria for AKI-2. After multivariable adjustment, patients meeting both urine output and creatinine criteria for AKI-1 were more likely to experience prolonged CICU stay (OR 4.9, 95%CI 3.3–7.4, *p* < 0.01) and more likely to require renal replacement therapy (OR 10.5, 95%CI 5.5–21.9, *p* < 0.01) than those meeting only the AKI-1 urine output criterion. Patients meeting both urine output and creatinine criteria for AKI-1 were at an increased risk of mid-term mortality compared to those diagnosed with AKI-1 by urine output alone (HR 2.8, 95%CI 1.6–4.8, *p* < 0.01). Patients meeting both urine output and creatinine criteria for AKI-2 were more likely to experience prolonged CICU stay (OR 16.0, 95%CI 3.2–292.0, p < 0.01) or require RRT (OR 11.0, 95%CI 4.2–30.9, p < 0.01) than those meeting only the urine output criterion. Patients meeting both urine output and creatinine criteria for AKI-2 were at a significantly increased risk of mid-term mortality compared to those diagnosed with AKI-2 by urine output alone (HR 3.6, 95%CI 1.4–9.3, *p* < 0.01).

**Conclusions:**

Patients diagnosed with the same stage of AKI by different KDIGO criteria following cardiac surgery have significantly different short and mid-term outcomes. The KDIGO criteria need to be revisited before they can be used to stratify reliably the severity of AKI in cardiac surgery patients. The utility of the criteria also needs to be explored in other settings.

**Electronic supplementary material:**

The online version of this article (10.1186/s12882-018-0946-x) contains supplementary material, which is available to authorized users.

## Background

Acute Kidney Injury (AKI) occurs in up to 50% of patients following cardiac surgery [1, 2]. Even in its mildest form, AKI is associated with increased mortality and prolonged Critical Care Unit stay [3–6]. AKI requiring renal replacement therapy (RRT) occurs in 2–5% of patients following cardiac surgery and is associated with mortality of up to 60% [1, 7, 8]. The Kidney Disease: Improving Global Outcomes (KDIGO) AKI guidelines were designed to standardise the criteria for AKI based on serum creatinine and urine output (Table 1) [9]. Patients are assigned the same stage of AKI regardless of which criteria (urine output, serum creatinine or both) for that stage are met. However, concerns have been raised that the guidelines’ urine output criteria are poorly calibrated [10].

**Table 1:** KDIGO criteria for diagnosis of AKI in adults [9]

Stage of AKI	Serum Creatinine	Urine output
1	1.5–1.9 times baseline OR ≥0.3 mg/dl (≥26.5 μmol/l) increase within 48 h	< 0.5 ml/kg/h for 6–12 h
2	2.0–2.9 times baseline	< 0.5 ml/kg/h for ≥12 h
3	≥3.0 times baseline OR Increase in serum creatinine to ≥4.0 mg/dl (≥353.6 μmol/l) OR Initiation of renal replacement therapy	< 0.3 ml/kg/h for ≥24 h OR Anuria for ≥12 h

Each stage of AKI is diagnosed when any of the criteria for that stage of AKI are met

Studies validating the KDIGO guidelines following cardiac surgery have frequently stratified patient risk based on serum creatinine alone as reliable urine output data are difficult to collect [1, 11–13]. Studies that have had access to urine output data have tended to be relatively small and have disagreed on the importance of urine output when identifying those at risk of adverse outcomes. [2, 14, 15]. A recent study with access to urine output data after cardiac surgery did demonstrate that patients with AKI diagnosed on oliguria alone had increased long-term mortality but due to the relatively small sample size they were unable to assess the importance of urine output within each AKI level [6].

The objective of this study was to validate the KDIGO guidelines for AKI by assessing the outcomes of patients meeting different criteria for each stage of AKI after cardiac surgery.

## Methods

### Data

**Fig. 1:**
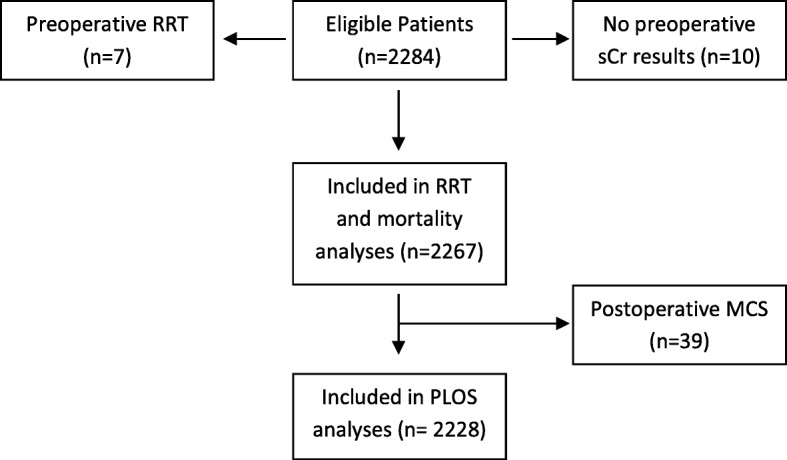
Flow chart for inclusion of patients in analyses. RRT = renal replacement therapy, sCR = serum creatinine result, MCS = mechanical circulatory support, PLOS = prolonged length of stay

Data from consecutive patients admitted to the cardiac intensive care unit (CICU) following cardiac surgery at Wythenshawe Hospital (part of Manchester University NHS Foundation trust) were collected prospectively between January 2013 and May 2015. Wythenshawe Hospital is a tertiary centre for adult cardiac surgery, cardiothoracic transplantation and mechanical circulatory support as a bridge to cardiac transplantation or recovery. Patients requiring RRT preoperatively and those with no preoperative creatinine values were excluded as shown in Fig. 1. Patients who received mechanical circulatory support were excluded from length of stay (LOS) analyses as their CICU stay was prolonged while awaiting definitive treatment. All data were collected as part of the Vascular Governance North West (VGNW) database and processed according this project’s protocols and ethical approvals.

Serum creatinine concentration was usually measured daily and all available results were extracted from the hospital’s pathology laboratory database. Our institution’s laboratory measures creatinine using techniques based on Jaffe chemistry with a total imprecision of < 6%. Every creatinine value for each patient was analysed and both relative and absolute increases in creatinine were used to classify AKI stages according to the KDIGO criteria (Table 1). The relative increases were calculated using the most recently recorded preoperative level as the baseline value. Urine output was recorded hourly on the CICU electronic patient record. Where the hourly value was recorded as none or zero, this value was accepted whereas when no value was entered for a given hour the next volume of urine recorded was divided equally by the number of blank hours prior to this recording. Whenever urine output fell below the thresholds in the KDIGO criteria, the time and appropriate stage of AKI was recorded. The need for RRT and postoperative LOS on CICU were identified from the electronic patient record. Serum creatinine concentration and urine output measurements recorded after initiation of RRT were not included in analyses as both are influenced heavily by RRT itself.

The hospital clinical governance database recorded 2-year all-cause mortality and the preoperative comorbidity, urgency and complexity of surgery as measured by the logistic EuroSCORE [16]. Prolonged LOS was defined as a CICU stay longer than 120 h for cardiac transplant patients or > 72 h for all other patients.

### Statistical analyses

Patients were assigned to groups based on the stages of AKI they reached according to the KDIGO guidelines. Within the groups that reached each AKI stage, patients were categorised as either i) meeting the urine output criteria ii) meeting the serum creatinine criteria or iii) meeting both urine output and serum creatinine criteria. Rates of prolonged LOS, RRT and 2-year mortality for those who did not develop AKI were compared with those for patients diagnosed with AKI-1 by urine output alone. Analyses within groups of patients meeting different combinations of criteria for each stage of AKI were then performed. The null hypothesis was that outcomes would be similar between patients diagnosed with the same stage of AKI based on the different KDIGO criteria.

Univariable analyses of categorical outcomes were performed using the chi-square test or Fisher’s exact test in the event of sparse data. The logistic EuroSCORE [16] which calculates mortality risk for cardiac surgery based on 13 preoperative variables (including preoperative renal function) and four operative variables was used to adjust for surgical risk in multivariable logistic regression models. The logistic EuroSCORE has been shown to have adequate discriminatory ability in UK cardiac surgery [17]. The results of the multivariable analyses are detailed in the Additional file 1.

Univariable and multivariable analyses of mid-term mortality rates were performed using the log-rank test and Cox proportional hazards regression modelling respectively. Data cleaning and statistical analyses were conducted using R (R Foundation for statistical computing) [18].

## Results

Data from 2284 patients were available. Seven patients who required RRT preoperatively and ten patients with no preoperative creatinine values were excluded leaving 2267 patients for the analysis (Fig. 1). Patient characteristics are shown in Table 2. There were 1448 patients who did not develop AKI during their CICU stay. A total of 819 (36.1%) developed AKI and 147 (6.5%) required RRT. There were 177 (7.8%) patients who died within two years of surgery. Of the 1448 patients who did not develop AKI, 255 (17.7%) had a prolonged LOS on CICU and the 2-year mortality rate for this group was 3.9%.

**Table 2:** Characteristics of patients admitted to the cardiac intensive care unit following cardiac surgery

Characteristic	All (*n* = 2267)	AKI-1UO (*n* = 370)	AKI-1 sCr (*n* = 192)	AKI-1 Both (*n* = 210)	AKI-2 UO (*n* = 97)	AKI-2 sCr (*n* = 92)	AKI-2 Both (*n* = 33)
Age, mean (sd), years	65.9 (11.6)	67.3 (10.5)	67.6 (12.7)	68.5 (11.8)	67.7 (10.9)	66.7(12.3)	64.4 (12.3)
Female gender, %	27.2	30.8	28.1	26.7	34.0	27.2	24.2
Weight, mean (sd), Kg	81.5 (15.8)	89.4 (16.6)	75.3 (14.0)	87.2 (17.7)	96.5 (17.3)	80.1 (16.3)	89.1 (18.0)
Logistic EuroSCORE, median (Interquartile range)	4.0 (2.1–7.7)	4.4 (2.5–7.7)	7.7 (3.5–18.1)	6.3 (2.8–13.0)	4.3 (2.5–7.6)	9.7 (4.2–18.4)	5.6 (2.3–15.6)
Operation, n (%)							
CABG	1211 (53.4)	177 (47.8)	56 (29.2)	92 (43.8)	49 (50.5)	28 (30.4)	10 (30.3)
Valve	477 (21.0)	93 (25.1)	50 (26.0)	39 (18.6)	26 (26.8)	23 (25.0)	8 (24.2)
CABG and Valve	301 (13.3)	60 (16.2)	41 (21.4)	39 (18.6)	13 (13.4)	12 (13.0)	5 (15.2)
Aortic	122 (5.4)	19 (5.1)	16 (8.3)	16 (7.6)	4 (4.3)	11 (12.0)	3 (9.1)
Cardiac Transplantation	53 (2.3)	5 (1.4)	19 (9.9)	10 (4.8)	1 (1.0)	8 (8.7)	4 (12.1)
MCS	39 (1.7)	6 (1.6)	8 (4.2)	6 (2.9)	2 (2.1)	4 (4.3)	2 (6.1)
Other – minor	20 (0.9)	3 (0.8)	1 (0.5)	5 (2.4)	0 (0.0)	3 (3.3)	1 (3.0)
Other – major	44 (1.9)	7 (1.9)	1 (0.5)	3 (1.4)	2 (2.1)	3 (3.3)	0 (0)
Urgency, n (%)							
Elective	1321(58.3)	217 (58.6)	99 (51.6)	110 (52.4)	56 (57.7)	42 (45.7)	14 (42.4)
Urgent	890 (39.3)	145 (39.2)	83 (43.2)	89 (42.4)	38 (39.2)	43 (46.7)	17 (51.5)
Emergency	40 (1.8)	7 (1.9)	8 (4.2)	7 (3.3)	3 (3.1)	5 (5.4)	1 (3.1)
Salvage	16 (0.7)	1 (0.3)	2 1.0)	4 (1.9)	0 (0.0)	2 (2.2)	1 (3.1)
CPB time, median (Interquartile range), minutes	101.0 (80.0–130.0)	100.0 (81.0–133.2)	129.0 (97.0–182.0)	107.5 (83.3–132.8)	99.0 (75.5–120.0)	112.0 (90.0–155.0)	123.0 (81.0–157.0)

*UO* urine output, *sCr* serum creatinine, *CABG* coronary artery bypass graft, *MCS* mechanical circulatory support, *CPB* cardiopulmonary bypass

### Acute kidney injury stage 1 (urine output only) vs no AKI

AKI-1 was diagnosed in 772 (34.1%) patients (Table 3) with 370 (47.9%) of these patients meeting only the urine output criterion (AKI-1-UO). As AKI-1-UO patients had the best outcomes (among patients who developed AKI) these patients were compared with the no AKI group. On univariable analysis, the rate of prolonged LOS for AKI-1-UO (39.6%) was significantly higher than for patients without AKI (*p* < 0.01). There were 22 (5.9%) AKI-1-UO patients who died within 2-years although this was not statistically significantly higher than the 2-year mortality rate in the no AKI group (*p* = 0.10). On multivariable analysis adjusted for the logistic EuroSCORE the risk of prolonged LOS for AKI-1-UO was higher (OR 2.8, 95%CI 2.2–3.6, *p* < 0.01) but the mortality risk within the first two years was not significantly higher (HR 1.4, 95%CI 0.9–2.3, *p* = 0.18) than for those without AKI.

**Table 3:** Influence of urine output and serum creatinine criteria for AKI-1 on outcomes

Criteria met	N (%)	Progressed to RRT, N(%)	Prolonged LOS N(%)	2-year mortality, N(%)
Urine output alone	370 (47.9)	16 (4.3)	145 (39.6)	22 (5.9)
Creatinine alone	192 (24.9)	28 (14.6) *	119 (64.7)*	24 (12.5)*
Urine output AND Creatinine	210 (27.2)	58 (27.6) *†	155 (76.0)* †	39 (18.6)*

*AKI* acute kidney injury, *RRT* renal replacement therapy, *LOS* length of stay

*= *p* values for comparison with urine output alone group < 0.01 on univariable analysis

† = *p* values for comparison with serum creatinine alone group < 0.02 on univariable analysis

### Acute kidney injury stage 1

Of the other patients diagnosed with AKI-1, 192 (24.9%) met only the serum creatinine concentration criteria (AKI-1-sCr) and 210 (27.2%) met both urine output and creatinine criteria (AKI-1-both). Details of the outcomes for these groups are shown in Table 3. On univariable analysis, rates of prolonged LOS and RRT were significantly higher for AKI-1-sCr than for AKI-1-UO patients (*p* < 0.01 for both). The 2-year mortality rate for AKI-1-sCr was also significantly worse than that for AKI-1-UO (*p* < 0.01). Outcomes for those with AKI-1-both were worse still with prolonged LOS and RRT rates significantly worse than those for the AKI-1-sCr group (*p* < 0.02 for both). The 2-year mortality rate for AKI-1-both was higher than that for AKI-1-sCr, but this difference did not achieve statistical significance (*p* = 0.09). The 2-year mortality rate for AKI-1-both was however significantly higher than that for AKI-1-UO (*p* < 0.01).

On multivariable analysis adjusted for the logistic EuroSCORE, when compared with AKI-1-UO, those with AKI-1-sCr had higher risks of prolonged LOS (OR 2.6, 95%CI 1.7–3.9, *p* < 0.01) and RRT (OR 3.2, 95%CI 1.5–7.2, *p* < 0.01). Similarly, compared with AKI-1-UO, AKI-1-both was associated with even greater risks of prolonged LOS (OR 4.9, 95% CI 3.3–7.4, *p* < 0.01) and RRT (OR 10.5, 95%CI 5.5–21.9, *p* < 0.01). Mortality risk within the first two years following surgery was greater for AKI-1-both than that for AKI-1-UO (HR 2.8, 95% CI 1.6–4.8, *p* < 0.01) but the smaller difference in mortality risk between AKI-1-sCr and AKI-1-UO over the same period (HR 1.4, 95%CI 0.7–2.7) was not statistically significant (*p* = 0.29).

### Acute kidney injury stage 2

There were 222 (28.8%) patients with AKI-1 who progressed to AKI-2. In 97 (43.7%) of these patients, AKI-2 was based on urine output alone (AKI-2-UO), in 92 (41.4%) it was based on serum creatinine concentration alone (AKI-2-sCr) and in 33 (14.7%) diagnosis was based on both criteria (AKI-2-both). Outcomes for these groups are shown in Table 4. On univariable analysis, the rates of prolonged LOS and RRT for AKI-2-sCr were significantly higher than for AKI-2-UO (*p* ≤ 0.01 for both) but the difference in 2-year mortality was not statistically significant (*p* = 0.20). Again, the rates of prolonged LOS and RRT for AKI-2-both were significantly higher than for AKI-2-sCr (*p* ≤ 0.02 for both) but the difference in 2-year mortality rates was not significantly different (*p* = 0.16). 2-year mortality in the AKI-2-both group was however significantly higher than that in the AKI-2-UO group (*p* = 0.01).

On multivariable analysis, compared with AKI-2-UO, AKI-2-sCr was associated with increased risks of prolonged LOS (OR, 2.1 95%CI 1.0–4.4, *p* = 0.04) and RRT (3.2, 95%CI 1.4–7.7, *p* < 0.01). Similarly compared with AKI-2-UO, AKI-2-both carried even greater risk of prolonged LOS (OR 16.0, 95% CI 3.2–292.0, p < 0.01) and RRT (OR 11.0, 95%CI 4.2–30.9, p < 0.01). Mortality during the first two postoperative years was significantly higher for AKI-2-both than AKI-2-UO (HR 3.6, 95% CI 1.4–9.3, p < 0.01) but the difference in mortality risk between AKI-2-sCr and AKI-2-UO was not statistically significant (HR 1.5, 95%CI 0.6–3.5, *p* = 0.40).

**Table 4:** Influence of urine output and serum creatinine criteria for AKI-2 on outcomes

Criteria met	N (%)	Progressed to RRT, N(%)	Prolonged ICU LOS, N(%)	2-year mortality, N(%)
Urine output alone	97 (43.7)	11 (11.3)	58 (61.1)	10 (10.3)
Creatinine alone	92 (41.4)	30 (32.6) *	70 (79.5)*	15 (16.3)
Urine output AND Creatinine	33 (14.9)	20 (60.6) *†	30 (97.0)* †	9 (27.3)*

*AKI* acute kidney injury, *RRT* renal replacement therapy, *LOS* length of stay

*= *p* values for comparison with urine output alone group ≤0.01 on univariable analysis

† = *p* values for comparison with serum creatinine alone group ≤0.02 on univariable analysis

### Acute kidney injury stage 3

AKI-3 by KDIGO criteria was diagnosed in 173 patients. In 47 (27.2%) of these patients, criteria for AKI-1 or AKI-2 had not been met before RRT was started almost immediately after surgery. There were 26 (15.0%) patients who met AKI-3 criteria based on urine output or creatinine without needing renal replacement therapy. In 16 of these patients, AKI-3 was based on a high serum creatinine levels with preserved urine output. For nine AKI-3 patients, anuria was observed for 12 h but only because the patient was not catheterised prior to discharge to the ward. 157 (85%) patients diagnosed with AKI-3 suffered a prolonged LOS. The small numbers within the subgroups precluded further analyses for AKI-3.

### Comparative survival analysis

**Fig. 2:**
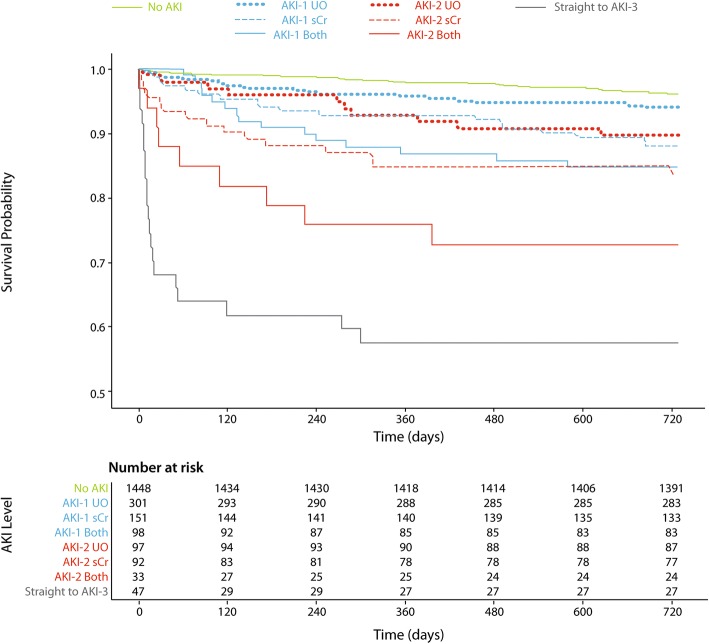
Kaplan Meier plots stratified according to the KDIGO criteria met for the maximum stage of AKI attained up to AKI-2. A separate group of patients who went straight to AKI-3 is shown for comparison. AKI – Acute Kidney Injury, UO – urine output, sCr – serum creatinine

To allow a visual comparison of survival between AKI stages rather than simply within AKI stages, patients were grouped according to the urine output and serum creatinine criteria met for the maximum stage of AKI attained by the patient (up to and including AKI-2). The group of patients who went straight to AKI-3 due to early initiation of RRT is also shown. The survival curves for these groups are shown in Fig. 2 which demonstrates clear overlap of the 2-year mortality risk for AKI-1 and AKI-2 patient subgroups.

## Discussion

This represents the first study to explore the associations between different criteria for multiple stages of KDIGO-defined AKI and morbidity and mortality after cardiac surgery. The frequency of AKI in our population was broadly similar to that previously reported in cardiac surgery patients [1, 19, 20]. RRT was more frequent than reported elsewhere [1, 6–8, 14] possibly as a substantial number of high risk tertiary referral, transplant and mechanical circulatory support patients are referred to our centre.

Univariable analyses identified stepwise increases in the risk of prolonged LOS, RRT and 2-year mortality within each stage of AKI where patients met urine output criteria, creatinine criteria or both. The increases in risk of poor outcomes were confirmed on multivariable analysis. This trend was previously reported in general ICU patients [21] and in cardiac surgery patients [6] although in the latter study the differences were not statistically significant.

In this study, when comparing outcomes for patients diagnosed with the same stage of AKI, all patients who met any criteria for that stage were included. This approach was chosen to improve the clinical relevance of our findings. In practice, clinicians cannot know whether a patient with AKI-1 will subsequently develop AKI-2 until the higher stage is diagnosed or the patient has left CICU without meeting AKI-2 criteria. As a consequence of this approach, some patients appear in both the AKI-1 and AKI-2 analyses. However, for comparison of survival outcomes between patients suffering different stages of AKI, each patient was placed retrospectively into a subgroup according to the maximum AKI stage reached during their CICU stay to avoid double counting.

The KDIGO AKI guidelines were developed using expert opinion at a conference of the Kidney Disease: Improving Global Outcomes group [9]. The guidelines drew on previous diagnostic criteria such as AKIN [22] and RIFLE [23] using urine output and creatinine to grade severity of renal dysfunction. The guidelines referenced several studies which demonstrated that patients with increasing stages of AKI had greater subsequent need for RRT and increased mortality risk. These findings were used to support the stratification of AKI within the KDIGO guidelines. However, none of the validation studies cited assessed the risk associated with AKI diagnosed by hourly urine output measurement [24–30]. The authors acknowledged that the urine output thresholds were less well substantiated than those related to serum creatinine concentration. Indeed, they noted that “the influence of urinary output criteria on AKI staging needs to be further investigated” and these concerns have been echoed in subsequent work validating the guidelines in their entirety [10]. The calibration of urine output thresholds might be particularly poor for cohorts such as those who have undergone major surgery for whom oliguria may be an appropriate response to surgery.

Many studies assessing the KDIGO guidelines in cardiac surgery patients used creatinine criteria alone to classify AKI and explore its association with subsequent adverse outcomes [12, 20]. Those which have included urine output disagreed on whether UO diagnosed by AKI was associated with adverse outcomes [2, 6, 14]. The current study contains more than three times the number of participants as the largest similar study into this topic in cardiac surgery patients. It is also the first to explore differences between with rates of adverse outcomes in those who meet different criteria within the same AKI stage for both AKI-1 and AKI-2.

Our findings support conclusions drawn from earlier studies that the risk of adverse outcomes associated with AKI diagnosed by UO alone is relatively low [2, 14]. In many cases the oliguria which resulted in diagnoses of AKI-1 and AKI-2 by urine output alone was almost certainly an appropriate physiological response to the stress of surgery. It is understandable that physiologically appropriate oliguria was associated with a prolonged LOS on CICU, but equally it is unsurprising that such oliguria was not associated with significantly increased 2-year mortality rates. In contrast, the risk of adverse outcomes in patients who met both urine output and creatinine criteria for AKI-1 or AKI-2 was markedly higher. Patients who suffered AKI-1 meeting both criteria had worse 2-year survival than those who suffered AKI-2 by urine output alone. This is highly relevant to treating clinicians; in these instances an AKI-1 diagnosis may be falsely reassuring. To ensure that the direct relationship between increasing AKI stages and risk of adverse outcomes is maintained, the AKI classification criteria may need to be adjusted to reduce the importance placed on isolated oliguria and increase the risk attributed to fulfilling both urine output and creatinine criteria. While we have studied patients undergoing cardiac surgery, similar findings may be reproduced in other cohorts, particularly those undergoing other types of major surgery and this should be the focus of further investigation.

### Study limitations

This study is based on consecutive data from a large tertiary cardiac surgery centre in the UK. The data utilised has been rigorously cleaned using reproducible algorithms. There were very few missing data and very few cases excluded from the analysis. Postoperative urine output and serum creatinine concentration data were available for every patient. Creatinine was measured on the first postoperative day for 2265 of the 2267 patients (99.7%) and while this proportion did decline gradually as the length of admission increased, even on the seventh postoperative day, 220 of the 240 (91.7%) patients who remained on CICU had a daily creatinine measurement. Urine output data was available for all patients throughout their admission.

Survival data was extracted from our hospital’s clinical governance database which is populated automatically by data from the NHS digital spine database. This approach is potentially less robust than using a dedicated follow-up process due to potential time delays in updating the status of the patient after death. However, as at least three months elapsed between the end of the follow up period for all patients and the extraction of survival data, this is unlikely to have affected the results.

We are confident that we have controlled for most relevant preoperative and intraoperative differences between the groups in our regression analyses by using the well validated logistic EuroSCORE. Although most known confounders including pre-operative renal function were included as variables in the logistic EuroSCORE, we cannot guarantee that all potential preoperative confounders have been adjusted for. Differences in post-operative management can have profound effects on urine output and serum creatinine. In addition, variations in post-operative management could influence the outcome measures utilised in this study. Adjusting for variations in post-operative management was beyond the scope of this study and is unlikely to have influenced the results as at the time of the study there were no institutional guidelines regarding management of AKI which would have led to systematic differences in treatments between the patient groups.

This study aimed to test the KDIGO criteria across the most heterogeneous sample possible. The cohort included those undergoing cardiac transplantation and those receiving mechanical circulatory support. Such patients are known to have greater risks of AKI, RRT and other adverse outcomes than the general cardiac surgery population. Patients from these higher risk groups made up 46%(*n* = 21) of those who started RRT prior to any urine output or creatinine criteria for AKI being met and this may have contributed to the high mortality rate associated in the “straight to AKI-3” group (Fig. 2).

## Conclusions

The current KDIGO guidelines assign the same AKI stage to patients with markedly different risks of adverse outcomes after cardiac surgery. This study identified a stepwise increase in the frequency of poor outcomes following cardiac surgery where urine output criteria, creatinine criteria or both criteria for each AKI stage were met. Moreover, 2-year mortality for some subgroups within AKI-1 clearly overlapped that of some subgroups within AKI-2. The KDIGO criteria need to be revisited before they can be used to reliably stratify the severity of AKI in cardiac surgery patients on the critical care unit. The applicability of these criteria to other patient groups within the critical care unit, particularly those in whom oliguria may be an appropriate physiological response also requires further evaluation.

## Additional file

Additional file 1: Tables detailing the logistic regression models used for the multivariable analyses described in the results section. (DOCX 20 kb)

## Abbreviations

AKI: Acute kidney injury
CICU: Cardiac intensive care unit
HR: Hazards ratio
KDIGO: Kidney Disease: Improving Global Outcomes
LOS: length of stay
OR: Odds ratio
RRT: Renal replacement therapy
sCr: Serum creatinine concentration
UO: Urine output
VGNW: Vascular Governance North West
