# Supplementary material for: The KDIGO acute kidney injury guidelines for cardiac surgery patients in critical care: a validation study
Source: BMC Nephrol. 2018 Jun 25;19:149. doi: 10.1186/s12882-018-0946-x (PMC6020229; doi:10.1186/s12882-018-0946-x)
Supplement: Supplementary file 1 — Tables detailing the logistic regression models used for the multivariable analyses described in the results section. (DOCX 20 kb) [file 12882_2018_946_MOESM1_ESM.docx]

Table A1- Multivariable logistic regression model for PLOS in group of patients with no AKI or AKI-1-UO

| Variable | Odds Ratio | 95% confidence interval | | P value |
| --- | --- | --- | --- | --- |
| (Intercept) | 0.15 | 0.12 | 0.18 | <0.01 |
| Logistic EuroSCORE | 1.06 | 1.04 | 1.08 | <0.01 |
| AKI-1-UO | 2.80 | 2.16 | 3.64 | <0.01 |

EuroSCORE- European System for Cardiac Operative Risk Evaluation; AKI-1-UO – acute kidney injury stage 1 by urine output

Table A2- Cox proportional hazards regression model for 2-year mortality in group of patients with no AKI or AKI-1-UO

| Variable | Hazard ratio | 95% confidence interval | | P value |
| --- | --- | --- | --- | --- |
| Logistic EuroSCORE | 1.05 | 1.03 | 1.06 | <0.01 |
| AKI-1-UO | 1.41 | 0.85 | 2.34 | 0.18 |

EuroSCORE- European System for Cardiac Operative Risk Evaluation; AKI-1-UO – acute kidney injury stage 1 by urine output

Table A3- Multivariable logistic regression model for PLOS in group of patients with AKI-1

| Variable | Odds ratio | 95% confidence interval | | P value |
| --- | --- | --- | --- | --- |
| (Intercept) | 0.51 | 0.40 | 0.66 | <0.01 |
| Logistic EuroSCORE | 1.03 | 1.01 | 1.05 | <0.01 |
| AKI-1-UO | 0.00 | - | - | - |
| AKI-1-sCr | 2.58 | 1.74 | 3.85 | <0.01 |
| AKI-1-both | 4.85 | 3.25 | 7.35 | <0.01 |

EuroSCORE- European System for Cardiac Operative Risk Evaluation; AKI-1-UO – acute kidney injury stage 1 by urine output, AKI-1-sCr– acute kidney injury stage 1by serum creatinine concentration, AKI-1-both – acute kidney injury stage 1 by urine output and serum creatinine concentration.

Table A4- Multivariable logistic regression model for RRT in group of patients with AKI-1

| Variable | Odds ratio | 95% confidence interval | | P value |
| --- | --- | --- | --- | --- |
| (Intercept) | 0.03 | 0.01 | 0.05 | <0.01 |
| Logistic EuroSCORE | 1.03 | 1.01 | 1.04 | <0.01 |
| AKI-1-UO | 0.00 | - | - | - |
| AKI-1-sCr | 3.19 | 1.46 | 7.23 | <0.01 |
| AKI-1-both | 10.54 | 5.51 | 21.93 | <0.01 |

EuroSCORE- European System for Cardiac Operative Risk Evaluation; AKI-1-UO – acute kidney injury stage 1 by urine output, AKI-1-sCr– acute kidney injury stage 1by serum creatinine concentration, AKI-1-both – acute kidney injury stage 1 by urine output and serum creatinine concentration.

Table A5- Cox proportional hazards regression model for 2-year mortality in group of patients with AKI-1

| Variable | Hazard ratio | 95% confidence interval | | P value |
| --- | --- | --- | --- | --- |
| Logistic EuroSCORE | 1.03 | 1.02 | 1.05 | <0.01 |
| AKI-1-UO | 0.00 | - | - | - |
| AKI-1-sCr | 1.42 | 0.75 | 2.70 | 0.29 |
| AKI-1-Both | 2.81 | 1.64 | 4.82 | <0.01 |

EuroSCORE- European System for Cardiac Operative Risk Evaluation; AKI-1-UO – acute kidney injury stage 1 by urine output, AKI-1-sCr– acute kidney injury stage 1by serum creatinine concentration, AKI-1-both – acute kidney injury stage 1 by urine output and serum creatinine concentration.

Table A6- Multivariable logistic regression model for PLOS in group of patients with AKI-2

| Variable | Odds ratio | 95% confidence interval | | P value |
| --- | --- | --- | --- | --- |
| (Intercept) | 1.26 | 0.77 | 2.08 | 0.26 |
| Logistic EuroSCORE | 1.03 | 0.99 | 1.08 | 0.14 |
| AKI-2-UO | 0.00 | - | - | - |
| AKI-2-sCr | 2.10 | 1.03 | 4.37 | 0.04 |
| AKI-2-both | 15.99 | 3.16 | 292.04 | <0.01 |

EuroSCORE- European System for Cardiac Operative Risk Evaluation; AKI-2-UO – acute kidney injury stage 2 by urine output, AKI-2-sCr– acute kidney injury stage 2 by serum creatinine concentration, AKI-2-both – acute kidney injury stage 2 by urine output and serum creatinine concentration.

Table A7- Multivariable logistic regression model for RRT in group of patients with AKI-2

| Variable | Odds ratio | 95% confidence interval | | P value |
| --- | --- | --- | --- | --- |
| (Intercept) | 0.11 | 0.05 | 0.22 | <0.01 |
| Logistic EuroSCORE | 1.01 | 0.98 | 1.04 | 0.62 |
| AKI-1-UO | 0.00 | - | - | - |
| AKI-1-sCr | 3.18 | 1.40 | 7.65 | <0.01 |
| AKI-1-both | 11.00 | 4.17 | 30.94 | <0.01 |

EuroSCORE- European System for Cardiac Operative Risk Evaluation; AKI-2-UO – acute kidney injury stage 2 by urine output, AKI-2-sCr– acute kidney injury stage 2 by serum creatinine concentration, AKI-2-both – acute kidney injury stage 2 by urine output and serum creatinine concentration.

Table A8- Cox proportional hazards regression model for 2-year mortality in group of patients with AKI-2

| Variable | Hazard ratio | 95% confidence interval | | P value |
| --- | --- | --- | --- | --- |
| Logistic EuroSCORE | 1.04 | 1.02 | 1.06 | <0.01 |
| AKI-1-UO | 0.00 | - | - | - |
| AKI-1-sCr | 1.46 | 0.60 | 3.51 | <0.01 |
| AKI-1-Both | 3.58 | 1.38 | 9.30 | <0.01 |

EuroSCORE- European System for Cardiac Operative Risk Evaluation; AKI-2-UO – acute kidney injury stage 2 by urine output, AKI-2-sCr– acute kidney injury stage 2 by serum creatinine concentration, AKI-2-both – acute kidney injury stage 2 by urine output and serum creatinine concentration.
